# Approximating Empirical Surface Reflectance Data through Emulation: Opportunities for Synthetic Scene Generation

**DOI:** 10.3390/rs11020157

**Published:** 2019-01-16

**Authors:** Jochem Verrelst, Juan Pablo Rivera Caicedo, Jorge Vicent, Pablo Morcillo Pallarés, José Moreno

**Affiliations:** 1 Image Processing Laboratory (IPL), Parc Científic, Universitat de València, 46980 Paterna, Valéncia, Spain; 2 CONACyT-UAN, Secretaría de Investigación y Posgrado, Universidad Autónoma de Nayarit, Ciudad de la Cultura Amado Nervo, Tepic CP. 63155, Nayarit, Mexico

**Keywords:** emulation, machine learning, interpolation, spectroscopy, scene simulation

## Abstract

Collection of spectroradiometric measurements with associated biophysical variables is an essential part of the development and validation of optical remote sensing vegetation products. However, their quality can only be assessed in the subsequent analysis, and often there is a need for collecting extra data, e.g., to fill in gaps. To generate empirical-like surface reflectance data of vegetated surfaces, we propose to exploit emulation, i.e., reconstruction of spectral measurements through statistical learning. We evaluated emulation against classical interpolation methods using an empirical field dataset with associated hyperspectral spaceborne CHRIS and airborne HyMap reflectance spectra, to produce synthetic CHRIS and HyMap reflectance spectra for any combination of input biophysical variables. Results indicate that: (1) emulation produces surface reflectance data more accurately than interpolation when validating against a separate part of the field dataset; and (2) emulation produces the spectra multiple times (tens to hundreds) faster than interpolation. This technique opens various data processing opportunities, e.g., emulators not only allow rapidly producing large synthetic spectral datasets, but they can also speed up computationally intensive processing routines such as synthetic scene generation. To demonstrate this, emulators were run to simulate hyperspectral imagery based on input maps of a few biophysical variables coming from CHRIS, HyMap and Sentinel-2 (S2). The emulators produced spaceborne CHRIS-like and airborne HyMap-like surface reflectance imagery in the order of seconds, thereby approximating the spectra of vegetated surfaces sufficiently similar to the reference images. Similarly, it took a few minutes to produce a hyperspectral data cube with a spatial texture of S2 and a spectral resolution of HyMap.

## 1 Introduction

The acquisition of spectroradiometric data and associated biophysical variables are an essential part of the development and validation of imaging spectroscopy vegetation products [1–3]. However, field data collection is an expensive and tedious job, typically requiring the organization of a dedicated field and flight campaign. Despite committed efforts to collect highly qualitative empirical datasets, their quality can only be assessed in the subsequent analysis. Often, it then appears that there is a need for additional gap-filling data collection, e.g., in case the collected data represent insufficient variability for a proper mapping validation [4]. Basically, two options occur to acquire additional data. The obvious option is returning to the field to collect new measurements. However, this is not always feasible: repeating a campaign is not only costly and time-consuming, it may also be that too much time has passed and the vegetation conditions have changed. It implies that an alternative way to collect extra data has to be considered. In this respect, the second option involves generating new data based on the already existing empirical data, e.g., by means of interpolation or extrapolation techniques. Evidently, this approach will never replace the collection of original field data, yet it can provide an adequate approximation and is without costs.

Interpolation of spectral data is a standard practice in image processing applications, and various interpolation techniques are commonly used, both for gridded (i.e., systematically ordered) and scattered (i.e., arbitrarily ordered) datasets [5]. When it comes to field spectral data, given its irregular nature, only scattered interpolation methods are possible. The most widespread method is linear interpolation because of its processing speed and accuracy [6,7]. Since interpolation in scattered datasets relies on triangulation of the input space, it requires large computer memory in high dimensionality of the input space and the method becomes computationally expensive. Another drawback of linear interpolation in scattered data is that it does not allow extrapolating outside the given parameter space. Other interpolation methods such as inverse distance weighting [8] can be used for extrapolating at the expense of accuracy.

As an alternative of classical interpolation techniques, in this work we propose to exploit *emulation* to produce new empirical-like spectral data. The principle of emulation is approximating the original model by a surrogate statistical learning model, also referred to as a meta-model, or *emulator* [9,10]. Essentially, an emulator functions as an interpolation method, but based on statistical learning principles. When an accurate emulator has been developed, it can then approximate the original model at a tiny fraction of the original speed [11–13]. The use of emulator deals with some advantages such as the use of a scattered input parameter space, making it more versatile than several advanced interpolation methods (e.g., piece-wise cubic splines and Sibson’s method) and allows both interpolation and extrapolation. An important question hereby arises whether emulators are able to compete with interpolation methods in generating spectral outputs, both in terms of accuracy and processing speed. This has been recently analyzed for the emulation of deterministic models, e.g., radiative transfer models (RTMs) [14]. In the latter work, emulation clearly outperformed interpolation in terms of accuracy and this at competitive speed. In the work of Verrelst et al. [13], RTM-based emulators are used for the generation of a synthetic hyperspectral data. The simulation of optical images can play key roles in the development of new instruments, the quantitative evaluation of algorithms and in the training of both image analysis software and human analysts [15–17]. The challenge in image simulation remains to simulate as realistically as possible without running into a tedious computational burden [17]. In this respect, an open question emerges: whether emulators can be used to replicate rather irregular empirical spectral data, i.e., as measured by a spectroradiometer. If so, it would become possible to render synthetic scenes that approximates a degree of realism as measured by a spectroradiometer.

This brings us to the following main objective: to analyze the ability of emulators as an alternative of classical interpolation methods for the production of empirical-like hyperspectral data. Sub-objectives were: (1) to compare emulation methods against interpolation methods validated against a part of a field dataset; (2) to use the most successful method to simulate a large empirical-like dataset; and (3) to analyze the feasibility of constructing synthetic hyperspectral airborne and spaceborne imagery. To address these objectives, a widely used empirical dataset was examined, i.e., SPARC (Spectra Barrax Campaign) [18]. This dataset consists of several field biophysical variables that were collected over multiple crop types. Simultaneously, an overpassing satellite CHRIS (Compact High Resolution Imaging Spectrometer) hyperspectral image was acquired and m a flight campaign was conducted with a HyMap airborne hyperspectral sensor, leading to associated spaceborne and airborne surface reflectance data. These labeled hyperspectral datasets were first analyzed with the purpose of clarifying the predictive power of emulators and interpolation methods. Synthetic empirical-like spectra were subsequently generated based on the best evaluated method. Eventually, new possibilities were explored to render synthetic hyperspectral imagery.

The remainder of this paper is arranged as follows. Section 2.1 introduces the interpolation and emulation methods, while Section 3 presents ESA’s SPARC dataset and gives the empirical setup. The results are presented in Section 4, followed by applications of hyperspectral imagery generation. A discussion on emulation opportunities for the rendering of synthetic imagery is provided in Section 5. Section 6 concludes the work.

## 2 Interpolation and Emulation

### 2.1 Interpolation

Starting with the interpolation theory, let us consider a *D*-dimensional input space *χ* from where we sample **x** ∈ *χ* ⊂ ℝ^*D*^ in which a *K*-dimensional object function **f**(**x**; *λ*) = [*f* (**x**; *λ*_1_),…, *f*(**x**; λ_*K*_)] : ℝ ↦ ℝ^*K*^ is evaluated. In the context of this paper, *χ* comprises the *D* input variables that control the behavior of the function **f**(**x**; *λ*), i.e., spectral output. Here, *λ* represents the wavelengths in the *K*-dimensional output space (for sake of simplicity, the wavelength dependency is omitted in the formulation, i.e., **f**(**x**; *λ*) ≡ **f**(**x**)). An interpolation, $\hat{\text{f}}\left(\text{x}\right)$, is therefore a technique used to approximate model simulations, $\text{f}(\text{x})=\hat{\text{f}}(\text{x})+\varepsilon$, based on the numerical analysis of an existing set of *nodes*, **f**_*i*_ = **f**(**x**_*i*_), conforming to a pre-computed dataset. The concept of interpolation has been widely used in remote sensing applications, including retrieval of biophysical parameters and atmospheric correction algorithms [6,19,20].

The following interpolation techniques are commonly used given scattered data:

- Nearest-neighbor: This is the simplest method for interpolation, which is based on finding the closest *node* **x**_*i*_ to a query point **x**_*q*_ (e.g., by minimizing their Euclidean distance) and associating their output variables, i.e.,$\hat{\text{f}}\left({\text{x}}_{q}\right)=\text{f}\left({\text{x}}_{i}\right)$. This fast method is valid for both gridded and scattered datasets. However, it produces discontinuities of the underlying model being interpolated.
- Piece-wise linear: This method is commonly used in remote sensing applications due to its balance between computation time and interpolation error. The implementation of linear interpolation is based on the Quickhull algorithm [21] for triangulations in multi-dimensional input spaces. For the scattered input data, the piece-wise linear interpolation method is reduced to finding the corresponding Delaunay simplex [22] (e.g., a triangle when *D* = 2) that encloses a query *D*-dimensional point **x**_*q*_ (see Equation (1)):
  (1) $${\hat{\text{f}}}_{i}\left({\text{x}}_{q}\right)=\overset{D+1}{\underset{j=1}{\sum^{\text{​}}}}\omega_{j}\text{f}\left(x_{j}\right),$$
  where *ω_j_* are barycentric coordinates of **x**_*q*_ with respect to the *D*-dimensional simplex (with *D* + 1 vertices) [23]. Since **f**(**x**) is a *K*-dimensional function, the result of the interpolation is also *K*-dimensional.

However, linear interpolation causes discontinuities on the first derivative of the interpolated model. In addition, in scattered datasets, the underlying Delaunay triangulation is computationally expensive in high dimensional input spaces (typically *D* > 6) and is also limited by its intensive memory consumption [21,24]. In practice, it implies that it cannot do extrapolation. To predict the missing samples, here linear interpolation is used in combination with the following method:

- Inverse Distance Weighting (IDW) [8]**:** Also known as *Shepard’s method*, this method weights the *n* closest nodes to the query point **x**_*q*_ (see Equation (2)) by the inverse of the distance metric *d*(**x**_*q*_, **x**_*i*_) : *χ* ↦ ℝ^+^ (e.g., the Euclidean distance):
  (2) $$\hat{\text{f}}\left({\text{x}}_{q}\right)=\frac{\sum_{i=1}^{n}\omega_{i}\text{f}\left({\text{x}}_{i}\right)}{\sum_{i=1}^{n}\omega_{i}},$$
  where *ω_i_* = *d*(**x**_*q*_, **x**_*i*_) ^−*p*^, and *p* (typically *p* = 2) is a tuneable parameter known as *power parameter*. When *p* is large, this method produces the same results as the nearest-neighbor interpolation.

The method is computationally cheap but it is affected by nodes far from the query point. The modified Shepard’s method [25] aims to reduce the effect of distant *grid points* by modifying the weights with Equation (3):

(3) $$\omega_{i}={\left(\frac{R-d\left({\text{x}}_{q},{\text{x}}_{i}\right)}{R\cdot d\left({\text{x}}_{q},{\text{x}}_{i}\right)}\right)}^{p},$$

where *R* is the maximum Euclidean distance to the *n* closest nodes.

### 2.2 Emulation

Emulation can essentially be considered as an interpolation technique, but then based on statistical learning principles [11–14]. The basic idea is that an emulator uses a limited number of simulator runs, i.e., input–output pairs (corresponding to labeled training samples), to infer the values of the complex simulator output given a yet-unseen input configuration. As with interpolation, once the emulator is built, it is not necessary to perform any additional runs of the model; the emulator computes the output that is otherwise generated by the simulator [9]. Note that building an emulator is in principle nothing more than building a statistical learning regression model as often done for biophysical variable retrieval applications, but in reversed order: whereas a retrieval model converts input spectral data (e.g., reflectance) into one or more output biophysical variables, an emulator converts input biophysical variables into output spectral data [11]. See also [26] for a systematic review on biophysical variable retrieval methods applicable to spectroscopy data.

When it comes to emulating spectral outputs, however, the challenge lies in delivering a full spectrum, i.e., predicting multiple spectral bands. It bears the consequence that the learning methods should be able to generate multiple outputs to be able to reconstruct a full spectral profile. This is not a trivial task. Only few regression models can deal with multiple outputs. However, training a complex multi-output statistical model with the capability to generate so many output bands would take considerable computational time and would probably incur a certain risk of overfitting because of model over-representation. A workaround solution had to be developed that enables the regression algorithms to cope with large, spectroscopy datasets. An efficient solution is to take advantage of the so-called *curse of spectral redundancy*, i.e., the Hughes phenomenon. Since spectroscopic data typically show a great deal of collinearity, it implies that such data can be converted to a lower-dimensional space through dimensionality reduction techniques. Accordingly, converting the spectral data into a limited set of components that preserve most of the spectral information content implies that the multi-output problem is greatly reduced [13]. Afterwards, the components can again be converted to spectral data with an efficient approximation.

The first step thus involves building a statistically-based representation (i.e., an emulator) of the field data using statistical learning from a set of training data points derived from runs of the actual model under study (nodes in the context of interpolation). These training data pairs should ideally cover the multidimensional input space using a space-filling algorithm. The second step uses the emulator previously built in the first step to compute spectral output. Based on the above literature review and earlier conducted emulation evaluation studies [11–13], the following three machine learning regression algorithms (MLRAs) serve as powerful methods to function as accurate emulators: (1) kernel ridge regression (KRR) [27]; (2) Gaussian processes regression (GPR) [28]; and (3) neural networks (NNs) [29]. A description of these algorithms can be found in earlier related works [11,30,31].

## 3 Description of Used SPARC Dataset and Experimental Setup

### 3.1 SPARC Dataset

As part of the SPARC campaign [18], the following biophysical variables were measured within a total of 108 Elementary Sampling Units (ESUs) for different crop types (garlic, alfalfa, onion, sunflower, corn, potato, sugar beet, vineyard and wheat): (1) leaf chlorophyll content (LCC); (2) leaf area index (LAI); (3) fractional vegetation cover (FVC); (4) biomass; (5) leaf water content (LWC); and (6) canopy water content (CWC) [18].

During the campaign a CHRIS satellite image and airborne hyperspectral airborne HyMap flight-lines were acquired for the study site, during the month of July 2003. CHRIS onboard PROBA satellite measures over the visible/near-infrared (NIR) spectra from 400 to 1050 nm. It can operate in different modes, balancing the number of spectral bands, size of the covered area, and spatial resolution because of onboard memory storage reasons [32]. CHRIS data were acquired in Mode 1 (62 bands, full spectral information, pixel size 34 m). The spectral resolution provides a bandwidth from 5.6 to 33 nm depending on the wavelength. The images were atmospherically corrected according to the method proposed in [33]. HyMap flew with a configuration of 125 contiguous spectral bands, spectrally positioned between 430 and 2490 nm. Spectral bandwidth varied between 11 and 21 nm. The pixel size at overpass was 5 m. The flight-lines were corrected for radiometric and atmospheric effects according to the procedures in [33]. Finally, from both images, a top-of-canopy reflectance dataset was prepared, referring to the center point of each ESU and their corresponding biophysical variables values. Accordingly, datasets of CHRIS and HyMaP spectra with labels of biophysical variables were created. Additionally, to account for spectral variability of non-vegetated surfaces, 29 bare soil spectra (with biophysical variables set to 0) were added so that a total of 130 samples was reached.

### 3.2 Experimental Setup

To ascertain that the predictive power of the interpolation and emulation methods, the labeled SPARC datasets were 80%/20% split into two parts: (1) a training-testing part (104 samples); and (2) a validation part (26 samples). The validation part serves as reference for all the methods. Then, the training dataset was again split into 80% training and 20% testing to test the emulation power of the three MLRAs. Based on earlier evaluation studies [11–14], the spectral training data were first converted with a PCA into 20 components. The interpolation methods do not require a training step, meaning that the entire original 80% was used as scattered dataset wherein the interpolation methods were applied.

The study was conducted in ARTMO’s Emulator toolbox [11]. This graphical user interface emulator toolbox provides various tools to develop, optimize and validate emulators. Multiple emulators can be developed. It then analyzes the validation accuracy of each emulator by calculating the root-mean-square-error (RMSE) and the normalized RMSE (NRMSE) (%) difference between emulated spectra and validation RTM spectra per wavelength and also averaged over the full spectral range. In this latest version (v. 1.09), the emulator toolbox has been expanded with new tools such as scene emulation and validation and the option to export an emulator outside the toolbox, which facilitates the interested user to repeat this study or conduct similar experiments for its own purposes. The complete analysis was done on a 64 bits Windows i7-4790CPU3-6GHz, 16GB RAM.

## 4 Results

### 4.1 Interpolation vs. Emulation

The performances of interpolation and emulation were validated against the 20% validation dataset for the CHRIS and HyMap datasets. Based on the calculated statistics and recorded run-time in Table 1, the following trends can be observed: (1) KRR and GPR emulation approximated the surface reflectance data considerably more accurately than the two interpolation methods, who perform similarly with linear interpolation performing slightly better than nearest interpolation. For both datasets, GPR emulation performed slightly superior than KRR. The performance of NN to reconstruct surface reflectance data tended to be more unstable; for the CHRIS dataset, the NN emulator performed similarly to the other emulators, while, for the HyMap dataset, the NN emulator performed on the same order as the interpolation methods. (2) All emulators produced spectral output multiple times faster than interpolation techniques. The gain in speed was on the order of 10–30 times for GPR, and 250–400 times for KRR.

**Figure 1:**
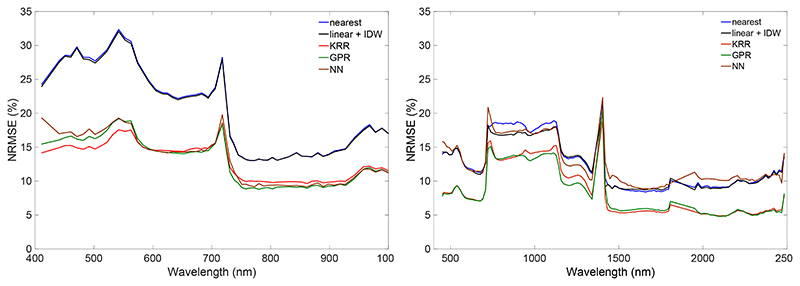
Wavelength-dependent NRMSE (%) results of the two interpolation methods and the three tested emulators, i.e., KRR, GPR, NN for CHRIS (**left**) and HyMap (**right**) datasets.

Probably a more comprehensive way to evaluate the predictive power of the interpolation and emulation methods is plotting the relative errors (NRMSE) as a function of wavelength (Figure 1). It can be noted that, for both the CHRIS and HyMap datasets, the emulation methods led to systematically lower errors. For the CHRIS dataset, both interpolation methods perform similarly, but that is not necessarily always the case, as shown for the HyMap dataset, and also tests with other datasets (results not shown). Lowest errors were obtained with the emulation methods KRR and GPR, with GPR producing slightly more accurate replications for the majority of wavebands. When inspecting the errors along the spectral range, some wavelength-dependent fluctuations can be observed, with most remarkable the peak in the HyMap dataset at 1404 nm. This band falls within the water absorption region, leading to a noisy dataset and thus more difficult to reproduce. However, this band is typically removed in vegetation applications (just as the water absorption bands in the 1900 nm region). A second HyMap peak with inaccuracies (at 723 nm) can also be observed in the CHRIS dataset (at 718 nm). These bands are located in the middle of the red edge, which is a highly dynamic narrow region where reflectance of vegetation changes rapidly from the visible to the NIR shoulder. Comparison of both datasets also reveals that the CHRIS dataset was less successful in replicating the spectra in the visible region than the HyMap dataset. While this suggests that the visible region of the satellite data is noisier than the airborne data, the key message is that the emulation methods are better able to cope with such data than the interpolation methods.

**Figure 2:**
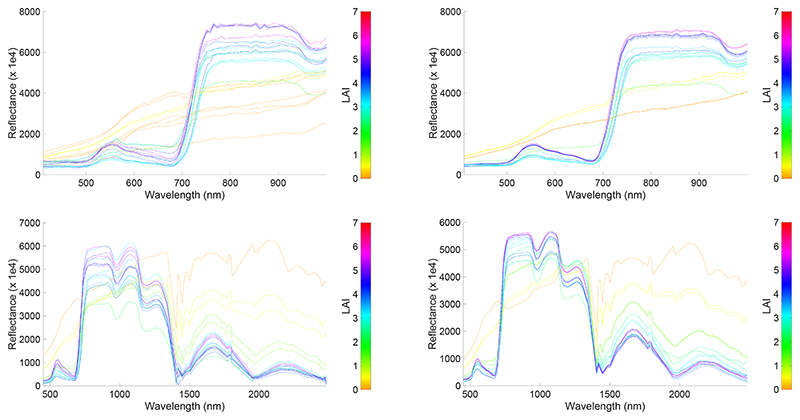
Original SPARC 20% validation spectra (**left**) and GPR-emulated spectra (**right**) for CHRIS (**top**) and HyMap (**bottom**) data, color plotted as a function of LAI.

Another way to evaluate the predictive power of the best-performing emulator is by comparing the GPR-emulated HyMap-like spectra against the original CHRIS and HyMap spectra (Figure 2). All six variables were sampled, although the spectra was color-scaled by LAI. The similarity between the original spectra and emulated spectra can be appreciated, although not all spectra were precisely replicated. That some differences appear is mainly due to the inclusion of bare soil spectra. Bare soil spectra are characterized by 0 values for all biophysical variables. An emulator is a deterministic model and thus generates only one spectral output when given 0 values for all variables. Hence, spectral variability over soil and man-made surfaces is lost.

**Figure 3:**
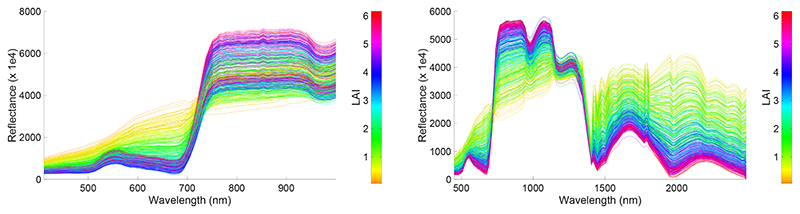
500 GPR-emulated CHRIS-like (**left**) and HyMap-like (**right**) surface reflectance spectra, color plotted as a function of LAI.

As a proof of concept and in an attempt to exemplify the predictive power of the GPR emulators, we generated 500 CHRIS-like and HyMap-like surface reflectance spectra based on random sampling of the six input variables. Although all six variables were randomly sampled, it took only 0.1 s to produce the associated output spectra. The obtained spectral variability can be viewed in Figure 3; the spectra is again color-scaled by LAI. With this figure, the potential of the emulator to rapidly generate sensor-specific hypespectral spectra of vegetated surfaces can be appreciated. Consequently, it should become similarly possible to emulate complete hyperspectral imagery.

### 4.2 Emulation of Hyperspectral Imagery

Having demonstrated the predictive power of emulators to produce empirical-like hyperspectral surface reflectance data, we then assessed whether emulators can be used to construct synthetic hyperspectral data cubes. Three demonstration cases are provided: (1) the emulation and validation of a synthetic spaceborne CHRIS image; (2) the emulation and validation of a synthetic airborne HyMap image; and (3) the emulation of a synthetic hyperspectral image with the spatial texture of Sentinel-2 (S2) and the spectral resolution of HyMap.

#### 4.2.1 CHRIS-Like Image

**Figure 4:**
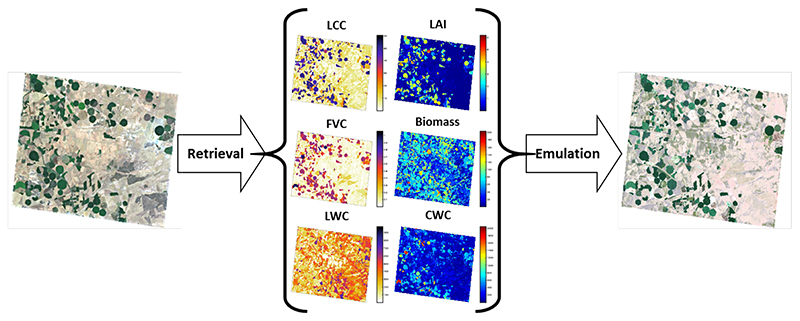
Schematic overview of RGB and emulated synthetic CHRIS image over agricultural site Barrax, Spain (R: 653 nm; G: 553 nm; B: 460 nm).

First, we assessed whether the above-developed emulator can also be used to reconstruct a synthetic CHRIS image (744 by 635 pixels). To do so, GPR retrieval models for all six SPARC variables (i.e., LCC, LAI, FVC, biomass, LWC and CWC) were developed and applied to a CHRIS image to obtain input maps (see [30,31] for details on GPR biophysical variables retrieval). As outlined in Figure 4, the following approach was pursued. The above-developed CHRIS emulators (KRR, GPR, and NN) were first run with inputs coming from the input maps. The emulated CHRIS-like image was then band-per-band compared against the reference CHRIS image.

**Figure 5:**
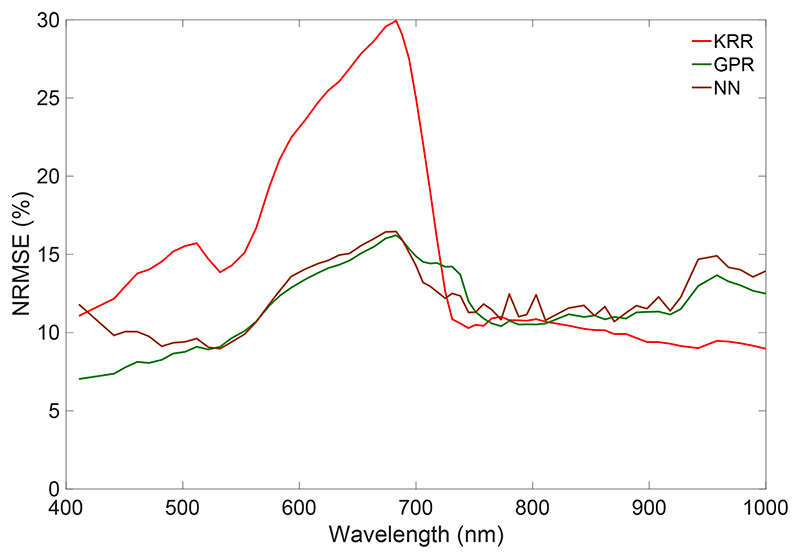
Wavelength-dependent NRMSE (%) comparison of scenes as generated by the three tested emulators (KRR, GPR, NN) against a reference CHRIS scene.

All three above-developed CHRIS emulators ran quickly in rendering the CHRIS-like image, with KRR and NN running extremely quickly, producing the data cube in about 15 s, and GPR running in about 24 s. Each emulated band was band-by-band compared for all image pixels against the reference image by calculating the NRMSE, which was then plotted for all bands (Figure 5). Overall, GPR performed most stably with errors around 10%, followed by NN that performed somewhat more unstably in the NIR. KRR largely failed in the visible but excelled after passing the red edge.

**Figure 6:**
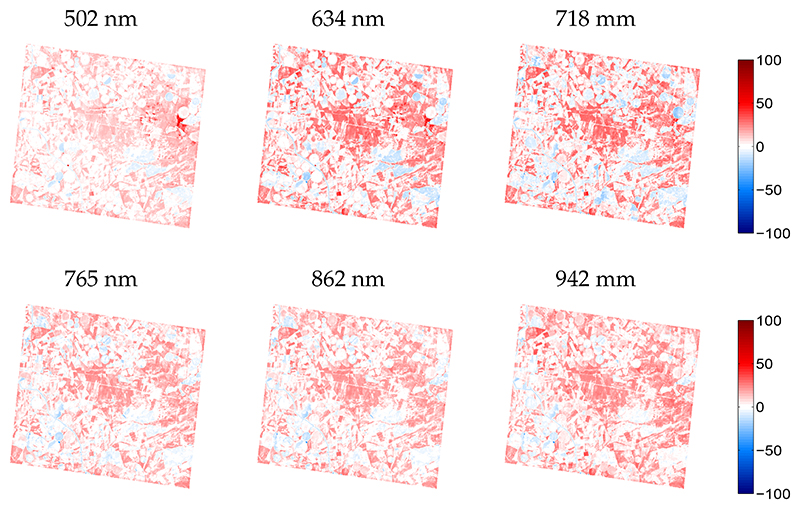
Relative difference maps (%) for arbitrarily chosen wavelengths between GPR-emulated scene and reference CHRIS scene.

To assess the quality of the GPR-emulated synthetic scene, relative errors maps are shown for six arbitrarily taken bands along the CHRIS spectral range in Figure 6—the other bands showed similar patterns (not shown for brevity). The whitish surfaces represent no differences, i.e., a perfect reconstruction of the pixel values, reddish colors signify an overestimation while bluish colors represent an underestimation. Substantial whitish areas along all spectral bands can be observed, meaning a perfect spectral reconstruction. This suggests that these areas have good to excellent approximations by the emulator. The underlying mechanism lies in that these areas were well represented by the SPARC training dataset. At the same time, some surfaces expose significant overestimations, especially around the irrigated agricultural fields. A closer look against the RGB image (see Figure 4) reveals that these areas are merely characterized by bare soils or fallow lands, areas that were hardly covered by the training dataset. Hence, this suggests that the emulator would benefit from the inclusion of a soil variable in order to be able deal with soil spectral variability.

#### 4.2.2 HyMap-Like Image

**Figure 7:**
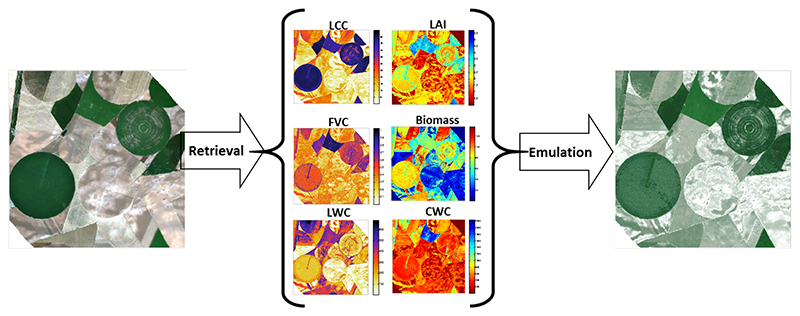
Schematic overview of RGB and emulated synthetic subset of HyMap scene over agricultural site Barrax, Spain (R: 646 nm; G: 555 nm; B: 462 nm).

**Figure 8:**
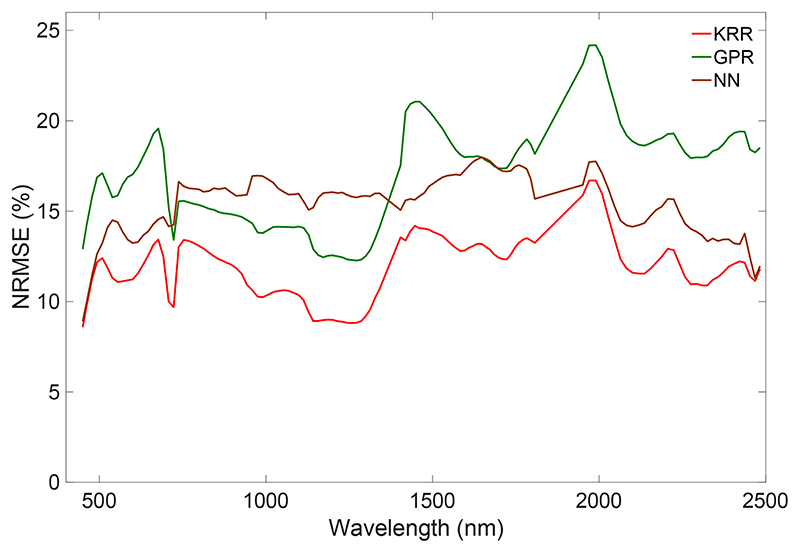
Wavelength-dependent NRMSE (%) comparison of scenes as generated by the three tested emulators (KRR, GPR, and NN) against a reference HyMap scene.

We subsequently assessed whether the HyMap emulators can be used to reconstruct a HyMap-like a subset of an HyMap image (500 by 500 pixels). Retrieval models for all six SPARC variables were again developed using GPR and applied an HyMap subset. As shown in Figure 7, the obtained maps were then used as input to run the three emulators. KRR again ran extremely quickly: it produced the data cube in less than 5 s. NN followed in about 12 s and GPR ran in about 18 s. KRR also appeared to replicate the reference image most accurately: KRR errors were on the order of 10–15% depending on the wavelength (Figure 8). Its combination of running quickly with high accuracy makes this KRR emulator attractive for further use.

**Figure 9:**
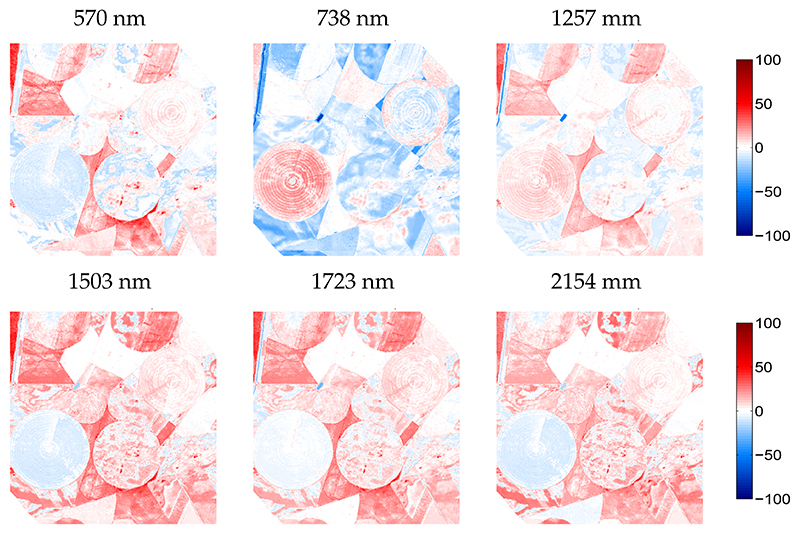
Relative difference maps (%) for arbitrarily chosen wavelengths between KRR-emulated scene and reference HyMap scene.

For the KRR-emulated HyMap scene, again some relative errors maps were given for six arbitrarily taken bands along the HyMap spectral range (Figure 9). The whitish surfaces along all spectral bands imply a perfect spectral reconstruction. For instance, the large circular agricultural parcel is mostly whitish or light bluish apart from the 738 nm bands. This suggests that these vegetated surfaces have good to excellent approximations by the emulator. Conversely, the spectral response of some surfaces exposed systematic overestimations, especially when moving towards the SWIR. A closer look against the RGB image reveals that these areas are merely characterized by bare soils or fallow lands, areas that were hardly represented by the training dataset. It again underlines that the emulator would benefit from the inclusion of a soil variable to deal better with soil spectral variability.

#### 4.2.3 Sentinel-2-Like Hyperspectral Image

**Figure 10:**
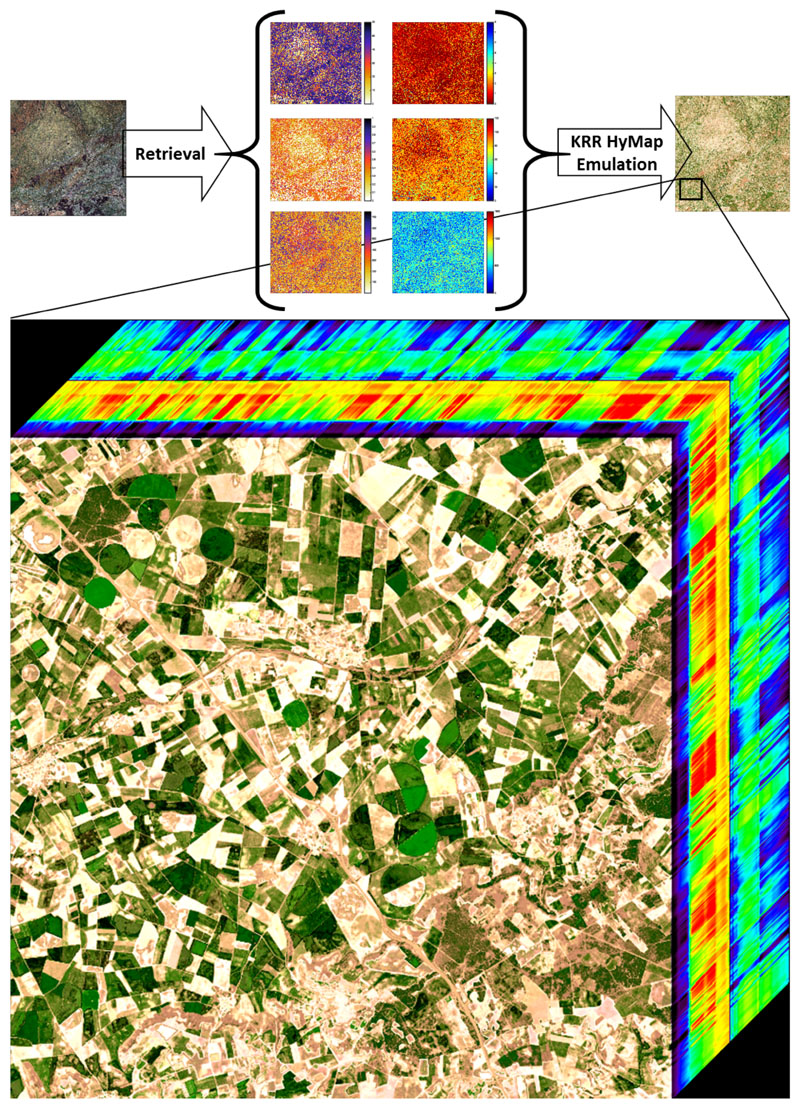
Schematic overview of RGB- and KRR-emulated synthetic hyperspectral S2-like image and data cube visualization of emulated subset over agricultural site Valladolid, Spain (R: 646.5 nm; G: 554.9 nm; B: 462.4 nm).

As a final scene rendering application, in this third experiment the KRR HyMap emulator was applied to generate a new synthetic hyperspectral image. Of interest is that the emulator technique enabled producing hyperspectral spectra based on inputs that come from anywhere, e.g., from biophysical variables derived from a routinely acquired satellite image. To exemplify this idea, we emulated a data cube with the spectral profile of HyMap and the spatial texture of a Sentinel-2 (S2). The pursued approach is as follows: first, biophysical variables maps coming from an original S2 image at 20 m resolution (5490 by 5490 pixels) were retrieved using GPR models. These maps subsequently served as input for the KRR HyMap emulator. It led to an emulated hyperspectral data cube with the spatial texture of S2 and the spectral richness of HyMap. It took 8 min to produce this hyperspectral S2-like image with a size of 29.4 GB. Because a single S2 image is rather big, Figure 10 displays a small subset of 700 × 700 pixels as a hyperspectral data cube so that the spatial details can be appreciated. The rendering of this subset took 4 s. The S2 spatial texture is clearly visible, with agricultural fields in pronounced green colors, and also spatial patterns of roads and riverbeds are easily observable. On the downside, since the emulator is based on training data over vegetated surfaces, it led to that the senescent or bare soil fields and other non-vegetated areas lack spectral variability, which can be observed by the homogeneous whitish fields.

## 5 Discussion

This study evaluated the use of statistical learning emulators to produce synthetic hyperspectral surface reflectance data similarly to how it would have been measured by a spectroradiometer. Emulation has been recently introduced as an attractive method to approximate the input–output functioning of deterministic models [10,11], and earlier proved to be successful in approximating full-spectrum RTM output data [12,13]. Here, it was assessed whether emulators can be developed to approximate sensor-specific empirical spectroradiometric data. Three emulation methods were compared against more conventional interpolation techniques, i.e., nearest neighbour and linear interpolation. Two empirical hyperspectral datasets with associated biophysical variables were analyzed: a spaceborne dataset, with surface reflectance measurements as acquired by a CHRIS overpass, and an airborne dataset, with surface reflectance measurements as acquired by HyMap. The emulators as developed by the machine learning regression algorithms KRR and GPR not only outperformed the tested interpolation techniques in terms of accuracy, but also produced the output spectra numerous times faster (up to 400 in case of KRR). This led us to suggesting that emulation is a more promising method than the commonly used interpolation methods in producing empirical-like synthetic surface reflectance data. However, it must be remarked that the used validation dataset was rather small (26 samples). The performances of all these methods will likely improve when having more samples available. This has been earlier tested with simulated data, where larger datasets clearly favoured the accuracies of all methods with superior results for emulators [14]. The performances of these methods when using large empirical datasets is left to be consolidated, yet the gain in speed as opposed to interpolation methods is clearly unsurpassable. To exemplify the emulators’ speed, 500 reflectance spectra were produced as a function of randomly combining input variables in less than a second. Another remark is that none of the tested methods performed equally stable along the spectral range; particularly, inaccuracies emerged in replicating noisy or highly dynamic spectral regions. This is not a surprise given that both interpolation or emulation methods develop a deterministic model and thus merely mimic general trends in the spectral data.

While the theoretical framework, strengths and weaknesses of interpolation versus emulation have been discussed before [14], here we address the potential of emulation for remote sensing applications. One attractive application of emulation is the rendering of synthetic passive optical imaging of the Earth’s surface. The rendering of synthetic images is one of the core elements in end-to-end satellite mission simulators [15,16,34]. Mission simulators are software tools used by scientists and engineers that allow: (1) consolidating the requirements of a satellite mission; and (2) testing and evaluating the performance of its instruments and data processing algorithms. Synthetic scenes therefore provide the reference bio-geophysical products maps to evaluate the performance of the mission as well as the input radiance observed by the instruments. The common approach is to use present reflectance maps from existing airborne/satellite images. This approach comes at the expenses of including instrumental noise to the reference scenes. The alternative approach is to use RTMs to propagate light though the surface and atmosphere. However, this comes at expenses of prohibitive computation time.

The emulated scenes presented in this paper show the capabilities of emulators to produce realistic scenes in terms of texture (by using external airborne/satellite imagery) and spectroradiometry (by training the emulator with labeled empirical spectra). The best-performing emulators were evaluated on their ability to reproduce CHRIS an HyMap data cubes by comparing against reference images. With KRR and NN emulators such scenes were generated quasi-instantly (in the order of seconds) and with wavelength-dependent relative errors below 15% when considering the whole scene. However, these errors varied largely depending on the spatial size (e.g., see CHRIS vs. HyMap) and within the scene depending on the land cover type. That emulation of HyMap led to local spots with high errors can also be interpreted in this light: a more diverse spectral variability is measured at a higher spatial scale, e.g., due to local variations in soil properties, which are not reproduced by the emulator. On the other hand, for vegetated surfaces a spectral variability similar as reference spectra was obtained both for CHRIS and HyMap. This is encouraging, given the fast processing and the low memory requirements of an emulator, i.e typically less than 1 MB. In principle, any kind of synthetic scenes with vegetated surfaces can be emulated, as long as input maps are available. This idea has been demonstrated with the rendering of a new hyperspectral image with the spatial texture of S2. These input maps can come from user-developed retrieval models, as done in this work, as well as from routinely acquire satellite products, or can be simulated, e.g., based on land cover maps and probability density functions [13,16]. Logically, the more realistic are the input maps, the more realistic are the emulated scene. In addition, processing speed can further be accelerated with more powerful computers. For instance, it took less than a minute (56 s) to repeat the rendering of the hyperspectral S2-like image on a newer PC (i7-8700CPU 3.70GHz, 32GB RAM).

Because of its versatility, the emulation technique opens up new opportunities in synthetic scenes generation. In the review paper by Han and Kerekes (2017) [17], four image simulation techniques have been reviewed with their pros and cons: (1) empirical approach; (2) image modification; (3) statistical approach; and (4) physical modeling. The usage of emulators for scene generation belongs to the category of the statistical methods. In statistical methods, Monte Carlo methods are of often used that generate individual pixels that conform to the statistical abundance data or machine learning to replicate real image phenomena [17]. It was also argued that the statistical models often lack the realism of empirical imagery that occurs given the complexity of the real world. With the emergence of emulators trained by empirical data—as has been demonstrated here—we believe progress has been made towards the statistical generation of realistic scenes.

Having outlined the potential of emulators for scene generation applications, it did not escape our attention that an emulator can only perform as well as the labeled training data. It is well understood that emulation never has the ambition to replace the need for field data collection (i.e., spectral observations and associated measured biophysical variables). Field data are mandatory to train the emulators, i.e., the quality of the emulator is only as good as the quality of the measurements. However, an emulator is non-stochastic, i.e., emulated output spectra behaves deterministically within the patterns of earlier-trained data [11,13]. Hence, good quality of training data is indispensable for the development of an accurate emulator. For an emulator trained by empirical data, evidently the inclusion of more biophysical variables and larger datasets will enhance its versatility. Regarding the empirical SPARC dataset, while multiple variables and many samples were collected, the dataset is not perfect as it contains value replications for each of the variables, i.e., the same variable value for multiple spectra. At the same time, the SPARC dataset—just as any empirical data—suffers from imperfections, e.g., due to undesired variability as introduced through imperfect measurements or through imperfections in the measurement devices. Hence, some degree of noise is unavoidable. Accordingly, the quality of statistical models can still be improved, not only with the addition of carefully taken samples, but also with ensuring the measurements contain a large variability of unique variables values. Related to this remark, it remains problematic that the developed emulators were unable to approximate non-vegetated surfaces. Given the absence of a variable that controls spectral variability over non-vegetated surfaces, it led that all these surfaces were emulated with the same soil spectral profile. This is a shortcoming for realistic scene generation of natural surfaces. However, in principle, this limitation can easily be mitigated when having a soil property variable available such as soil moisture data [35]. Apart from data quality, another path where further emulation accuracy improvements may be encountered is in the used algorithm. Latest statistical learning algorithms can be very powerful, particularly of interest is the rapidly evolving field of deep learning [36]. Although advanced NN designs are mostly used into classification studies, deep learning methods such as transfer learning are increasingly used in regression, and could thus be exploited for emulation purposes (e.g., [37]).

Beyond the here presented demonstration cases, emulators can easily be developed and imported into other image processing applications with ARTMO’s Emulator Toolbox [11]. In the toolbox, emulators can be developed either based on RTM data or on empirical data, or a mixture of both. They can then be imported into various toolboxes including scene simulation. Simulated scenes through emulation can be either based on external input maps of biophysical variables or inputs can be also be based on user-defined land cover classes with associated variables and probability density functions [13]. Other ARTMO toolboxes that enable the running of emulators include: (1) global sensitivity analysis, allowing to identify the driver variables [12]; and (2) inversion of RTMs. Inversion is typically done by means of look-up tables, e.g., for atmospheric correction and retrieval or for the retrieval of vegetation variables. By replacing a computationally expensive RTM with its emulated counterpart, the inversion becomes extremely quick and therefore attractive for image processing.

Altogether, emulation can serve as a convenient technique to generate quickly a massive amount of synthetic spectral data that behave similarly to data observed by a sensor based on input biophysical variables. As such, it enables rendering scenes of vegetated surfaces with low computational cost. Moreover, in principle, multiple emulators can be combined in the rendering of synthetic scenes, e.g., specific emulators for different land cover classes such as a vegetation emulator, water emulator and an emulator that accounts for spectral variability of bare soil and man-made surfaces. Likewise, emulators can also be combined with the more sophisticated RTMs. For instance, the vegetated surfaces can be simulated by means of a canopy RTM while water bodies can be emulated, or the other way around, depending on the application and the preferred trade-off between required accuracy and processing speed. It is anticipated that future image simulation systems will offer blends of physical and statistical image simulation techniques that can be customized depending on the user’s requirements and preferred trade-off between accuracy and speed.

## 6 Conclusions and Outlook

This study demonstrated that emulation of empirical surface reflectance data labeled with biophysical variables offers a fast and convenient technique to generate an unlimited amount of empirical-like synthetic surface reflectance data. Emulators are statistical models that approximate spectral outputs as a function of input biophysical variables. While machine learning regression algorithms (MLRAs) have earlier been shown successful in emulating deterministic models, in this work, we analyzed whether statistical learning models can be used to emulate empirical spectral data. To analyze their ability to replicate surface reflectance data, three different MLRAs were compared against common interpolation methods for two empirical hyperspectral datasets. Emulation simulated surface reflectance data multiple times faster and also more precise than interpolation. This technique opens the door to rapidly produce empirical-like synthetic hyperspectral data, e.g., for the production of synthetic imagery. The running of emulators to render scenes has been demonstrated with the production of a synthetic CHRIS, HyMap and a new hyperspectral Sentinel-2-like image. Images were rendered instantaneously and particularly over vegetated surfaces sufficient realism as compared to reference images was preserved. Emulation can be concluded as a fast and easy alternative to simulate synthetic imagery, e.g., in preparation of future imaging spectroscopy missions. It is expected that in the near future emulators will find their way into end-to-end satellite mission simulators. For instance, exploratory efforts are underway to introduce emulators into the FLEX E2E simulator [15,16] for rendering more realistic FLEX-like reflectance and fluorescence imagery.

## Figures and Tables

**Table 1:** Interpolation vs. emulation validation results and CPU time for CHRIS (**top**) and HyMap (**bottom**) SPARC datasets.

Model	RMSE	NRMSE (%)	CPU (s)
**CHRIS**			
**Interpolation:**			
- nearest	653.3	20.7	0.1881
- linear + IDW	649.4	20.5	0.3040
**Emulation:**			
- KRR	436.3	13.0	0.0007
- GPR	420.6	13.0	0.0096
- NN	432.5	13.4	0.0070
**HyMap**			
**Interpolation:**			
- nearest	405.4	12.5	0.1501
- linear + IDW	398.2	12.2	0.2428
**Emulation:**			
- KRR	269.6	8.5	0.0006
- GPR	267.2	8.4	0.0086
- NN	412.0	12.6	0.0059
